# No major effects of vitamin D_3_ (1,25 dihydroxyvitamin D_3_) on absorption and pharmacokinetics of folic acid and fexofenadine in healthy volunteers

**DOI:** 10.1007/s00228-016-2050-0

**Published:** 2016-03-29

**Authors:** Gerd A. Kullak-Ublick, Christoph Gubler, Katharina Spanaus, Manfred G. Ismair, Tatiana Claro da Silva, Alexander Jetter

**Affiliations:** Department of Clinical Pharmacology and Toxicology, University Hospital Zurich, University of Zurich, Rämistrasse 100, CH-8091 Zürich, Switzerland; Department of Gastroenterology and Hepatology, University Hospital Zurich, University of Zurich, Zürich, Switzerland; Department of Clinical Chemistry, University Hospital Zurich, University of Zurich, Zürich, Switzerland; XenoGesis Ltd, Nottingham, UK

**Keywords:** Absorption, 1,25-dihydroxyvitamin D_3_, Fexofenadine, Folic acid, PCFT, OATP1A2

## Abstract

**Purpose:**

In Caco-2 cells, folate uptake via the proton-coupled folate transporter (PCFT) increases significantly by a 3-day treatment with 1,25-dihydroxyvitamin D_3_ (1,25(OH)_2_D_3_). Additionally, mRNA content and protein expression of the transporter OATP1A2 were increased up to ninefold with 1,25(OH)_2_D_3_. We investigated whether these in vitro findings can be confirmed in humans in vivo.

**Methods:**

Ten healthy volunteers (six women) received 5 mg folic acid orally once before and once together with the last intake of a 10-day course of 0.5 μg 1,25(OH)_2_D_3_ orally. One hundred twenty milligrams fexofenadine, an OATP1A2 substrate, was taken in 1 day before the first folic acid intake, and again on the ninth day of 1,25(OH)_2_D_3_ intake. Duodenal biopsies were taken for transporter mRNA assessments once before and once on the ninth or tenth day of the vitamin D_3_ course. Serum folic acid and fexofenadine concentrations were quantified with a chemiluminescence immunoassay and LC-MS/MS, respectively. Pharmacokinetics were compared between periods with standard bioequivalence approaches.

**Results:**

While geometric mean folic acid AUC_0-2h_, which mainly reflects absorption, was 0.403 and 0.414 mg/L·h before and after the vitamin D_3_ course (geometric mean ratio (GMR), 1.027; 90 % confidence interval (90 % CI), 0.788–1.340), the geometric mean fexofenadine AUC_0-2h_ was 1.932 and 2.761 mg/L·h, respectively (GMR, 1.429; 90 % CI, 0.890–2.294). PCFT- and OATP1A2-mRNA expressions in duodenal biopsies were essentially unchanged.

**Conclusions:**

No significant changes in folic acid and fexofenadine absorption were observed after a 10-day course of 1,25(OH)_2_D_3_ in humans in vivo. This study underlines the importance of confirming in vitro findings in vivo in humans.

## Introduction

The active form of vitamin D, 1,25-dihydroxyvitamin D_3_ (1,25(OH)_2_D_3_), exerts most effects, e.g., on calcium homeostasis, via binding to vitamin-D receptors (VDR) in the cell nucleus. As a heterodimer with the retinoid-X-receptor α (RXR), VDR regulates the transcription of numerous genes [1], like cytochrome P450 24A1, which is involved in 1,25(OH)_2_D_3_ biodegradation, tryptophan hydroxylase 2 in the brain [2], the organic anion transporting polypeptide 1A2 OATP1A2 [3], or the folate uptake transporter PCFT [4]. However, evidence for the role of 1,25(OH)_2_D_3_ in the functional regulation of these proteins usually comes from in vitro or animal experiments, and the relevance in humans is unknown.

The water-soluble vitamin folic acid is the precursor for the production of the metabolically active folates, which are essential for the synthesis of nucleic acids in human cells. Naturally occurring folates in food are conjugated to a polyglutamyl chain which is removed by the enzyme folate conjugase in the mucosal cells before the folate monoglutamates can be absorbed in the duodenum and jejunum [5] mainly by the PCFT (gene code *SLC46A1*) and, to a lesser extent, by the reduced folate carrier (RFC, gene code *SLC19A1*), which has a lower affinity for folic acid [6]. Absorption across the intestinal epithelium is the limiting step for the availability of diet-derived folates [7].

Tissues with a high turnover rate like bone marrow, lymphatic tissue, hair follicles, and tumor cells are most susceptible to folate deficiencies [8]. During pregnancy, neural tube defects such as spina bifida, and in non-pregnant persons, anemia and pancytopenia, a reduced immune response, and other pathologies may result. The total body folate store is thought to be 5–10 mg, of which 50 % is contained in the liver. Since folate supply in a western-style diet is often not sufficient to cover the daily requirement of 200–400 μg, nutrition is also fortified with folic acid. Foods rich in folic acid include meat (e.g., liver), legumes (e.g., lentils, soy products), fruits and vegetables (e.g., fennel, nuts, spinach, broccoli, cabbage, beetroot, potato), and starches (wholegrain bread, wheat bran, rice).

PCFT is an electrogenic transporter located in the apical enterocyte membrane of the proximal part of the small intestine and works best at low pH values [4, 9]. Mutations in the gene coding for PCFT were found in patients with hereditary folate malabsorption [10]. In cell culture experiments, it was shown that the gene coding for PCFT is transactivated by the VDR and that rat duodenal Pcft mRNA levels as well as PCFT mRNA levels and folate uptake in Caco-2 cells were dose-dependently increased during treatment with 1,25(OH)_2_D_3_ [4].

There are numerous other intestinal transporters for nutrients, ions, water, peptides, and also xenobiotics. Two important representatives of the organic anion transporting polypeptide family, OATP1A2 (gene code *SLCO1A2*) and OATP2B1 (gene code *SLCO2B1*), are expressed in several tissues, notably at the blood brain barrier, in cholangiocytes, in the apical domain of tubular cells of the distal nephron, and at the apical membrane of small intestinal epithelial cells. Although the expression level of OATP1A2 in human intestine is still controversial [11–13], OATP1A2 and OATP2B1 are responsible for the uptake of a number of drugs such as fexofenadine, rosuvastatin, and atenolol, but also for the transport of endogenous substrates such as bile acids, conjugated sex steroids, thyroid hormones, prostaglandin E_2_, etc. On the other hand, apical and basolateral intestinal efflux transporters like P-glycoprotein (P-gp, gene code *ABCB1*) and multidrug-resistance-related protein 3 (MRP3, gene code *ABCC3*), respectively, also exert influences on the absorption of drugs like fexofenadine [14]. In vitro data from our laboratory indicate that the expression of OATP1A2 is induced up to ninefold by 1,25(OH)_2_D_3_ [3], whereas nothing is known about an effect of 1,25(OH)_2_D_3_ on OATP2B1. Studies in vitro suggest that intestinal P-gp is upregulated by 1,25(OH)_2_D_3_ [15, 16], while reports on MRP3 are equivocal [15, 17].

These in vitro findings suggest that intestinal folate uptake and uptake of OATP1A2 substrates can be increased by 1,25(OH)_2_D_3_. However, the extent to which these in vitro findings are relevant in vivo in humans is unknown. The aim of this study, therefore, was to investigate the influence of a sufficiently long 1,25(OH)_2_D_3_ administration on the pharmacokinetics of folic acid and fexofenadine as marker substrates of PCFT and OATP1A2 activities, respectively, in healthy human volunteers. To investigate the link between pharmacokinetics and transporter expression and activities, we additionally quantified mRNA of PCFT, OATP1A2, OATP2B1, P-gp, and MRP3 in duodenal biopsies.

## Methods

### Study design and population

The study was performed as a prospective single arm, single center, pharmacokinetic study in 10 healthy volunteers at the Department of Clinical Pharmacology and Toxicology, University Hospital of Zurich, Switzerland. Subjects were eligible if they were between 18 and 65 years of age, had a body mass index between 18 and 30 kg/m^2^, and were in a good healthy condition, as determined by physical examination and laboratory assessment conducted prior to the study. The intake of concomitant medication was not allowed, except oral contraceptives in women. Participants were instructed not to take in any nutrition supplement, especially folic acid or food fortified with folic acid.

The study was approved by the Ethics Committee of the Canton of Zurich, Switzerland (KEK-ZH-no. 2012–0051), and by the Swiss National Health authority, Swissmedic. The clinicaltrials.gov identifier is NCT01856348. Written informed consent was obtained from each volunteer before any study related procedures were carried out. The study was carried out in accordance with the Declaration of Helsinki, amendment of Seoul, South Korea, 2008, and all pertinent guidelines and laws in force in Switzerland.

### Study procedures

On study day 1, 120 mg fexofenadine (Telfast®, Sanofi-Aventis, Meyrin, Switzerland), and on study day 2, 5 mg folic acid (Acidum folicum Streuli®, Streuli, Uznach, Switzerland) was given to the participants orally together with 200 mL light sparkling water. An esophago-gastro-duodenoscopy with biopsies from the duodenum to assess PCFT, OATP1A2, OATP2B1, P-gp, and MRP3 mRNA expression was performed on study day 1 or 2, depending on the availability of the endoscopy unit.

During the following 7 days (study days 3 to 10), participants were requested to take in 0.5 μg 1,25(OH)_2_D_3_ (Rocaltrol®, Roche Pharma, Reinach, Switzerland) daily in the morning at home and to fill in a diary for treatment adherence, adverse event reporting, and nutrition assessment. On study days 11 and 12, a second dose of 120 mg fexofenadine and 5 mg folic acid, respectively, was given together with the daily dose of 1,25(OH)_2_D_3_, and a second endoscopy was carried out.

All drug administrations on study days 1, 2, 11, and 12 were supervised by study personnel, and a mouth check was carried out. Before the study drug intakes on days 1, 2, 11, and 12, participants fasted for at least 1 h. Subjects consumed a light breakfast after the 2-h blood sampling and a lunch at least 6 h after study drug intakes on the pharmacokinetics days.

Serial blood samples to assess pharmacokinetics of fexofenadine (pre-dose and at 1, 2, 4, 6, 8, 10, and 24 h after fexofenadine intake) and folic acid (pre-dose and at 0.5, 1, 1.5, 2, 4, and 6 h after folic acid intake) were obtained on study days 1 (fexofenadine), 2 (folic acid), 11 (fexofenadine), and 12 (folic acid). Blood samples for the quantification of pre-dose 1,25(OH)_2_D_3_ serum concentrations were obtained on study days 1, 2, 11, and 12.

### Sample analysis

Blood samples were drawn from an indwelling catheter in the cubital vein and centrifuged at 4 °C and 3000 rpm for 10 min. The resulting serum was frozen at −80 °C until analysis. Concentrations of folic acid and 1,25(OH)_2_D_3_ in serum were measured at the Institute of Clinical Chemistry of the University Hospital Zürich, Switzerland, and fexofenadine was analyzed by XenoGesis Ltd, Nottingham, UK.

### Quantification of folic acid in serum

Folic acid was quantified in serum using a chemiluminescence immunoassay on the Access 2 immunoanalyzer (Beckman Coulter, Brea, CA, USA). This test is standardized to the World Health Organization International Standard 03/178.

In this assay, folic acid in the sample competes with a conjugate of folic acid and alkaline phosphatase for binding sites on a folic acid-binding protein. After immobilization of the resulting complex on a solid phase and removal of unbound material, addition of a chemiluminescent substrate induces a light signal that is inversely proportional to the concentration of folic acid in the sample.

The detection limit of this test is <1 μg/L and the limit of quantification <2 μg/L as indicated by the manufacturer. Inter- and intra-assay imprecision has been found to be lower than 10 % for concentrations >3.6 μg/L. Since the dynamic measurement range of this test is between 1.0 and 24.8 μg/L, many of the samples had to be diluted to allow measurement of folic acid concentrations higher than 24.8 μg/L. As recommended by the manufacturer, the Access Folate Calibrator S0 (zero) was used for the dilutions.

### Quantification of fexofenadine in serum

Fexofenadine (FEX) and fexofenadine-d6 (FEX-d6) were purchased from Sequoia Research Products Ltd. (Pangbourne, UK) and Santa Cruz Biotechnology (Heidelberg, Germany), respectively. HPLC grade methanol and formic acid were from Fisher Scientific (Loughborough, UK). Water for HPLC was purified on a Milli Q system (Millipore, Watford, UK).

FEX serum concentrations were assayed by LC-MS/MS using FEX-d6 as internal standard. FEX and FEX-d6 stock solutions were prepared at a concentration of 1 mg/mL in DMSO and stored at −20 °C. Independent weighings were used for standards and quality controls (QC). Working solutions were prepared by dilution of stock solutions in methanol. Standards and QCs were prepared by spiking 10 μL methanolic working solution into 50 μL blank human serum and adding 40 μL methanol plus 150 μL methanol containing 100 nM FEX-d6 as internal standard. Serum concentration curves were prepared at 2, 5, 10, 25, 50, 100, 250, 500, 1000, and 2000 ng/mL. QC samples were prepared in the same way containing 16, 160, and 1600 ng/mL FEX.

To each 50 μL serum sample, 50 μL methanol and 150 μL methanol containing 100 nM FEX-d6 as internal standard were added. Blank serum samples were prepared in the same way. All samples, standards, and QCs were vortex mixed and kept at −20 °C for a minimum of 2 h to allow complete protein precipitation. Samples were then centrifuged at 2500×*g*, 4 °C for 20 min, and supernatants were transferred for analysis by LC-MS/MS.

Five microliters of each sample was analyzed in MRM mode on a Waters Premier XE tandem quadrupole mass spectrometer using electrospray ionization and a Waters Acquity UPLC system (Waters Corp., Milford MA, USA). High performance liquid chromatography was performed on a ACE Excel 2 C18-AR, 50 × 2.1 mm column (ACE Ltd., Aberdeen, UK) using water (A) and methanol (B) both containing 0.1 % *v/v* formic acid as mobile phases. Flow rate was 0.5 mL/min, and the column temperature was 50 °C. A solvent gradient was used: solvent B increased from 5 % in the beginning to 95 % at 1.5 min, rose to 99 % from 1.55 till 2 min, returned to 5 % at 2.05 min, and remained stable until the next injection started.

MS/MS transitions were *m*/*z* 502.2 → 171.1 and 508.2 → 177.1 for FEX and FEX-d6, respectively. Dwell time was 50 ms, cone voltage was 46 V, and collision energy was 34 eV for both analytes. Peak area ratios of FEX and FEX-d6 were used for quantification. The lower and upper limits of quantification were 2 and 2000 ng/mL, respectively.

### Quantification of 1,25(OH)_2_D_3_ in serum

After manual immunoextraction of 1,25(OH)_2_D_3_ from the serum sample using a monoclonal antibody, the concentration of this parameter was determined with a commercially available 1,25(OH)_2_D_3_ assay on an immunoanalyzer (IDS-iSYS immunoanalyzer, IDS plc, Boldon, UK) using a competitive immunoassay method. 1,25(OH)_2_D_3_ from the serum sample competed with a 1,25(OH)_2_D_3_-acridinium conjugate for binding to a biotinylated anti-1,25(OH)_2_D_3_-antibody. After immobilization and washing of the resulting antigen-antibody-complex, light signal induced by a chemiluminescence reaction was measured.

Limit of detection of this test was 6.5 ng/L and limit of quantification, defined in accordance with the manufacturer’s recommendations as the concentration which can be measured with an inter-assay coefficient of variation not higher than 20 %, was 12.2 ng/L. Measurement range of this test was between 6.5 and 210 ng/L; thus, 1,25(OH)_2_D_3_ concentrations could be quantified in all samples without further dilution. Intra-assay imprecision has been found to be lower than 10 % at concentrations of 37.9 and 82.3 ng/L (4.6 and 9.7 %, respectively), inter-assay precision was 12.6 % at 34.9 ng/L and 11.9 % at a 1,25(OH)_2_D_3_ concentration of 81.9 ng/L.

### Transporter mRNA quantification in duodenal biopsies

Duodenal biopsies were immediately snap-frozen in liquid nitrogen and stored at −80 °C. Using the “Nucleospin miRNA” kit (Macherey-Nagel, Oensingen, Switzerland), frozen biopsies were immersed in the appropriate buffer and homogenized with a 1.5-mL pestle (VWR, Radnor, PA, USA), and RNA was extracted following the manufacturer’s instructions. Extracted RNA was subsequently analyzed by real-time PCR (RT-PCR) using TaqMan probes (Thermo Fisher, Waltham, MA, USA), and results were normalized to the enterocyte-specific housekeeping gene villin. Biopsies were identified according to subject (number) and whether they were collected before or after 1,25(OH)_2_D_3_ administration. All mRNA quantifications were done in triplicate.

### Pharmacokinetic analysis

A non-compartmental pharmacokinetic approach was chosen. The maximum serum concentration after drug intake (*C*_max_) and the time to reach *C*_max_ (*t*_max_) was directly taken from the plasma concentration-time data. The terminal elimination half-life (*t*_1/2_) was calculated by dividing ln(2) by the terminal elimination rate constant λ*z*, which was estimated by linear regression of ln-concentration data of the terminal part of the concentration-time curve. The area under the concentration-time curve (AUC) was calculated using the log-linear trapezoidal rule, either from the time of administration to a specified time point thereafter, or to the last quantifiable time point (AUC_0-last_). To obtain the AUC from administration extrapolated to infinity (AUC_0-∞_), the estimated AUC_last-∞_, which was calculated as *C*_last_ / λ*z*, was added to AUC_0-last_. The parameters *C*_max_ and AUC_0-2_ were selected for the characterization of the absorption phase of fexofenadine and folic acid.

### Statistical analysis

Since the effect of a 1,25(OH)_2_D_3_ administration on the pharmacokinetics of fexofenadine and folic acid, respectively, has not yet been investigated in humans, the minimally relevant difference necessary for proper sample size calculation could not be estimated on a scientific basis. However, if an increase in exposure towards fexofenadine, or folic acid, of 50–80 % is deemed relevant (corresponding to a “moderate” change in exposure according to FDA recommendations [18]), and if an inter-individual variability of roughly 20–30 % is imputed (coefficients of variation in folic acid AUC_0-∞_ after a single oral dose of 5 mg were around 25 % [19], and the coefficient of variation for fexofenadine AUC_0-∞_ after a single oral dose of 130 mg is reported to be 28.4 % [20]), a sample size of 10 participants would be sufficient to detect this difference with an *α* of 0.05 and a *β* of approximately 0.2.

For the comparison of pharmacokinetics of fexofenadine and folic acid, respectively, before and after 10 days of 1,25(OH)_2_D_3_ intake, an ANOVA-based relative bioequivalence assessment was carried out. The pharmacokinetic parameters of fexofenadine and folic acid before the start of intake of 1,25(OH)_2_D_3_ were taken as reference for these calculations. Point estimates of the geometric means ratios of the pharmacokinetic parameters of fexofenadine and folic acid during 1,25(OH)_2_D_3_ intake over the pharmacokinetic parameters of fexofenadine and folic acid before the 1,25(OH)_2_D_3_ dosing period were calculated alongside with their corresponding 90 % confidence intervals. An influence of 1,25(OH)_2_D_3_ administration on the pharmacokinetics of fexofenadine, or folic acid, was accepted as absent if the 90 % confidence intervals of the point estimates fell entirely within the bioequivalence zone of 0.8–1.25.

## Results

A total of 10 healthy volunteers (6 females, 4 males, mean age 26 ± 2 years, mean body mass index 22.9 ± 3.1 kg/m^2^, all non-smokers) were enrolled and completed the study. All adverse events were mild and transient. There was no statistically significant change in serum 1,25(OH)_2_D_3_ concentrations before and on the 11th and 12th study day: the geometric mean 1,25(OH)_2_D_3_ concentrations (geometric coefficient of variation) were as follows: day 1, 18.7 ng/mL (49 %); day 2, 19.1 ng/mL (48 %); day 11, 20.9 ng/mL (44 %); and day 12, 20.5 ng/mL (41 %).

**Fig. 1:**
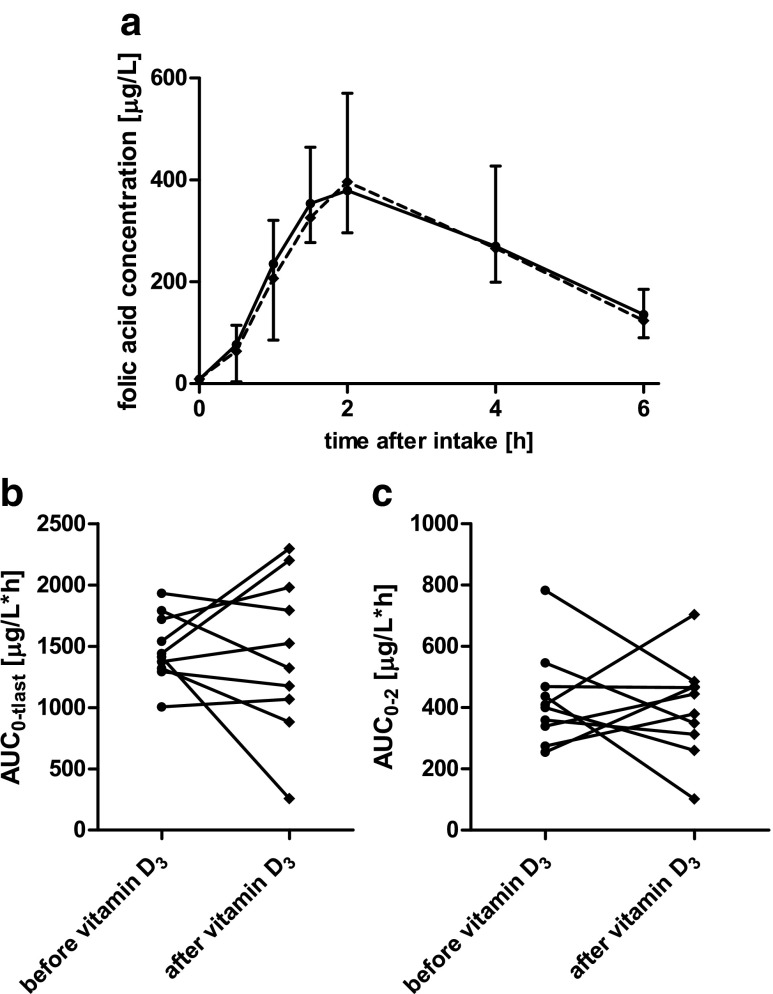
Mean folic acid serum concentration-time curves (**a**), AUC_0-last_ (**b**), and AUC_0-2_ (**c**) before (*circles*) and after (*diamonds*) a 10-day course of 0.5 μg of 1,25(OH)_2_D_3_ daily intake in 10 healthy male and female volunteers

**Fig. 2:**
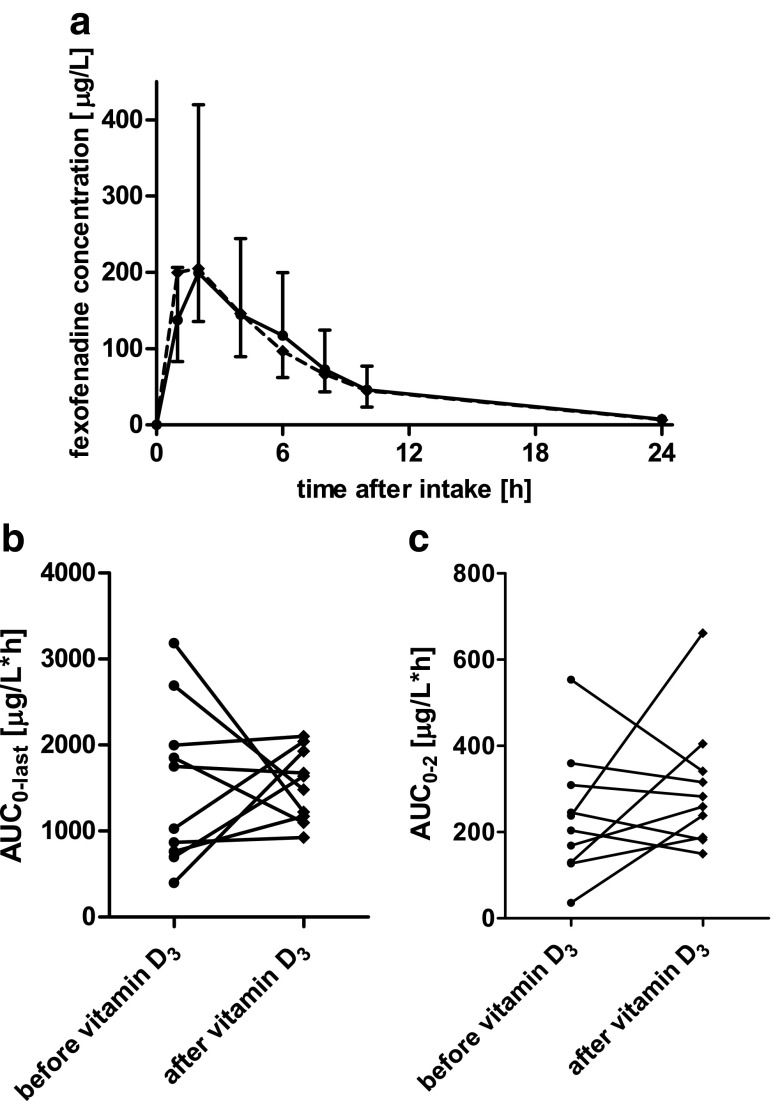
Mean fexofenadine concentration-time curves (**a**), AUC_0-last_ (**b**), and AUC_0-2_ (**c**) before (*circles*) and after (*diamonds*) a 10-day course of 0.5 μg of 1,25(OH)_2_D_3_ daily intake in 10 healthy male and female volunteers

Pharmacokinetics of folic acid and fexofenadine in serum before and after a 10-day course of oral 1,25(OH)_2_D_3_ are shown in Tables 1 and 2, respectively. Folic acid concentrations in one participant were much lower in all samples taken after 1,25(OH)_2_D_3_ intake, which was implausible so that these volunteer’s data were excluded from the pharmacokinetic and statistical analysis for folic acid. Mean concentration-time curves as well as individual changes in total AUC and AUC_0-2_, a parameter mainly reflecting absorption, are shown for folic acid and fexofenadine in Figs. 1 and 2, respectively. In summary, folic acid pharmacokinetics were not statistically significantly different after 10 days of 1,25(OH)_2_D_3_ intake (Table 1). For fexofenadine pharmacokinetics, statistically significant changes were also not observed (Table 2). Although the parameters of fexofenadine best reflecting absorption, i.e., *C*_max_ and AUC_0-2_, showed mean increases by 31 and 43 %, respectively, the inter-individual variabilities were too large to yield significant findings.

**Table 1:** Pharmacokinetics of folic acid in nine healthy volunteers before and after a 10-day course of 1,25(OH)_2_D_3_

	Before 1,25(OH)_2_D_3_	After 1,25(OH)_2_D_3_	Geometric mean ratio	90 % confidence interval
AUC_0-6_ [μg/L·h]	1465.5 (20.1 %)	1506.9 (34.7 %)	102.8	86.4–122.4
AUC_0-∞_ [μg/L·h]	2007.4 (24.8 %)	1967.6 (39.1 %)	98.0	82.7–116.2
AUC_0-2_ [μg/L·h]	403.1 (35.6 %)	414.0 (29.6 %)	102.7	78.8–134.0
*C*_max_ [μg/L]	407.7 (16.9 %)	416.8 (32.9 %)	102.2	83.9–124.5
*t*_max_ [h]	1.9 (0.8)	1.9 (0.2)	n.c.	n.c.
*t*_1/2_ [h]	2.7 (0.5)	2.5 (0.6)	n.c.	n.c.

Pharmacokinetic parameters are shown as geometric means (geometric coefficient of variation) for concentration-dependent parameters and as arithmetic mean (standard deviation) for *t*_max_ and *t*_1/2_

*n.c.* not calculated

**Table 2:** Pharmacokinetics of fexofenadine in 10 healthy volunteers before and after a 10-day course of 1,25(OH)_2_D_3_

	Before 1,25(OH)_2_D_3_	After 1,25(OH)_2_D_3_	Geometric mean ratio	90 % confidence interval
AUC_0-24_ [μg/L·h]	1263.8 (75.8 %)	1474.9 (30.5 %)	116.7	78.4–173.8
AUC_0-∞_ [μg/L·h]	1313.6 (73.9 %)	1521.3 (29.9 %)	115.8	78.4–171.0
AUC_0-2_ [μg/L·h]	193.2 (91.2 %)	276.1 (45.9 %)	142.9	89.0–229.4
*C*_max_ [μg/L]	169.8 (90.1 %)	222.5 (40.7 %)	131.0	82.1–209.1
*t*_max_ [h]	1.9 (1.4)	2.1 (1.0)	n.c.	n.c.
*t*_1/2_ [h]	5.2 (0.8)	4.9 (0.7)	n.c.	n.c.

Pharmacokinetic parameters are shown as geometric means (geometric coefficient of variation) for concentration-dependent parameters and as arithmetic mean (standard deviation) for *t*_max_ and *t*_1/2_

*n.c.* not calculated

**Fig. 3:**
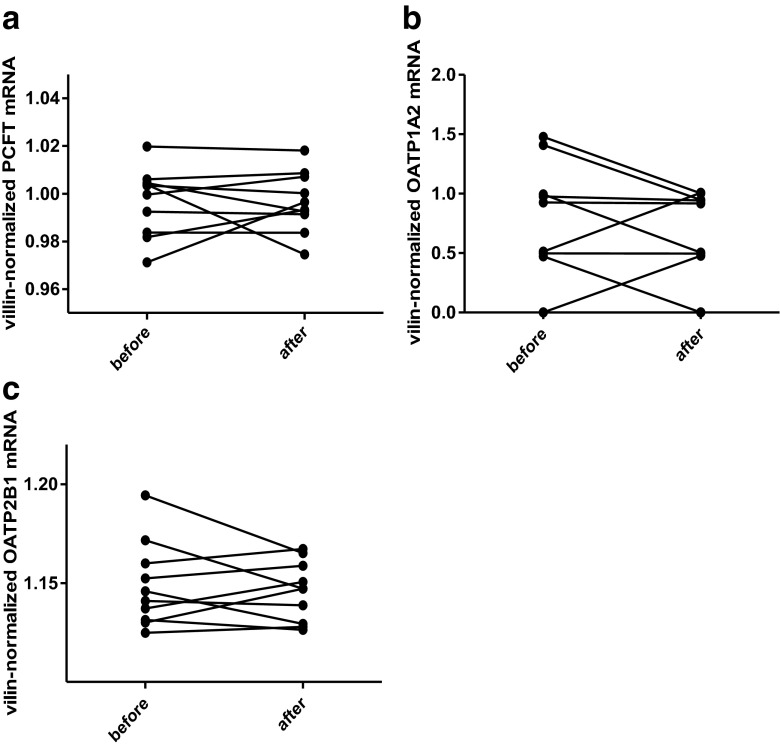
Villin-normalized PCFT (**a**), OATP1A2 (**b**), and OATP2B1 (**c**) mRNA levels in duodenal biopsies taken before and on the 9th or 10th day of (after) a 10-day course of 0.5 μg of 1,25(OH)_2_D_3_ daily intake in 10 healthy male and female volunteers

The villin-normalized mRNA contents of PCFT, OATP1A2, and OATP2B1 in the duodenal biopsies taken before and at the end of a 10-day course of 1,25(OH)_2_D_3_ are shown in Fig. 3. OATP1A2 mRNA could not be quantified in four samples from three volunteers. OATP2B1-, PCFT-, P-gp, and MRP3-mRNA were quantifiable in all biopsy samples. In an ANOVA-based evaluation, there was no statistically significant difference in geometric mean villin-normalized mRNA content of any of the transporters between the two biopsy time points, except for MRP3, where a marginally significant decrease in mRNA was observed. For OATP1A2, the geometric mean relative villin-normalized mRNA contents was 0.811 in biopsies taken before, and 0.727 in biopsies taken after 10 days of 1,25(OH)_2_D_3_ intake (geometric means ratio (GMR), 89.66; 90 % confidence interval (90 % CI), 65.87–122.03). For OATP2B1, the geometric mean mRNA contents were 1.149 before and 1.146 after the 10-day course (GMR, 99.75; 90 % CI, 98.96–100.54), and for PCFT, values were 0.997 before and 0.996 after the 1,25(OH)_2_D_3_ intake (GMR, 99.99; 90 % CI, 99.16–100.84). For the efflux transporter P-gp, the geometric mean mRNA contents were 1.860 before and 1.608 after the 1,25(OH)_2_D_3_ intake (GMR, 86.41; 90 % CI, 72.01–103.70), and corresponding values for MRP3 were 2.988 and 2.724 (GMR, 91.15; 90 % CI, 83.65–99.33).

## Discussion

This study aimed at clarifying whether an inducing effect of 1,25(OH)_2_D_3_ on folate uptake via PCFT could be confirmed in vivo in humans, as well as on the transport capacity of OATP1A2 and OATP2B1, because inducing effects on PCFT and OATP1A2 have been demonstrated in cell culture experiments [3, 4]. We could rule out that a 10-day course of 1,25(OH)_2_D_3_ has an effect on folate uptake and on PCFT expression in healthy human volunteers. Although the mean results for fexofenadine favored an increase in fexofenadine uptake of about 30–40 % after 10 days of 1,25(OH)_2_D_3_, these results were not statistically significant and the intestinal mRNA expression of OATP1A2 and OATP2B1 was not changed.

Two intestinal uptake mechanisms exist for folates in humans. While the PCFT is the most relevant transport mechanism, the RFC does not seem to play an important role in folate uptake in the intestines [6]. As experiments from our group have shown earlier, PCFT can be dose-dependently transactivated by binding of 1,25(OH)_2_D_3_ to a VDR-RXR heterodimer, which then binds to the PCFT promoter and roughly doubles PCFT expression and folate uptake in Caco-2 cells in vitro [4]. However, the present study shows that this effect is not quantifiable in vivo. In a post-hoc evaluation of a study which investigated the effects of different vitamin D supplementations on 25-hydroxyvitamin D (25(OH)D) concentrations in humans, others have recently found out that, although 25(OH)D concentrations rose after an 8-week supplementation course, no differences in folic acid plasma concentrations could be substantiated [21]. In our present study, we did not find an effect on PCFT mRNA expression in intestinal biopsies, which corroborates the absence of an effect of 1,25(OH)_2_D_3_ on folic acid absorption in vivo in humans. These results underline the importance of verifying in vitro findings by in vivo studies, although the explanations for the observed discrepancies are not obvious. It can be speculated that the 1,25(OH)_2_D_3_ concentration changes in the enterocytes (and the 1,25(OH)_2_D_3_ doses used) were too low to lead to a quantifiable effect on PCFT expression and activity. In vitro, a 3-day treatment with only 25 nM 1,25(OH)_2_D_3_ (corresponding to approx. 10 μg/L) was sufficient to show a significant PCFT mRNA increase in Caco-2 cells, but more pronounced effects were seen with 100–200 μg/L 1,25(OH)_2_D_3_ [4]. Trough serum concentrations (taken 24 h after the last intake) of at least 10 μg/L were present in 9 out of the 10 volunteers already at the start of the study and, as expected, did not change throughout the study. This finding is in line with the fact that 1,25(OH)_2_D_3_ serum concentrations are tightly controlled in the human body. It may be possible that higher doses would have led to a quantifiable effect. However, since higher doses of 1,25(OH)_2_D_3_ may confer an undue risk for prolonged hypercalcemia and related adverse effects, we did not select higher doses for the present study.

Most drugs which are absorbed from the intestine via drug transporters are not specific substrates for a single transporter. This is also the case with fexofenadine, which is taken up from the gut lumen by OATP1A2 and OATP2B1 [22], and is transported outwards towards the gut lumen by P-gp [23], while MRP3-mediated transport is directed towards the portal blood [13]. It is therefore not directly possible to unequivocally identify the mechanisms that ultimately determine absorption pharmacokinetics of fexofenadine after a 10-day course of 1,25(OH)_2_D_3_. OATP1A2 has been shown to be upregulated in vitro up to ninefold by a 3-day treatment of Caco-2 cells with a large dose of 500 nM (approx. 200 μg/L) 1,25(OH)_2_D_3_ for 24 h [3]. Additionally, it was also shown that P-gp mRNA and protein expression was increased in Caco-2 cells after 24 h treatment with 100 nM 1,25(OH)_2_D_3_, and that this effect diminished with time [15]. In vitro studies on the effect of 1,25(OH)_2_D_3_ on MRP3 led to contradictory results [15, 17]. The marginally significant decrease in MRP3 mRNA we observed here would even suggest a decrease in fexofenadine bioavailability, but the small extent of the change does not suggest any relevance. Since there is still controversy whether OATP1A2 is expressed in human intestine (we observed OATP1A2 mRNA in 16 out of 20 duodenal biopsies), since nothing is known about the inducibility of OATP2B1 by 1,25(OH)_2_D_3_, and since we did not observe any relevant changes in the duodenal mRNA of the four transporters involved in fexofenadine pharmacokinetics, there is no mechanistic explanation which would support the non-significant trend towards slightly higher mean fexofenadine exposure after a 10-day treatment with 1,25(OH)_2_D_3_ observed in the present study in healthy volunteers. Taken together, a 10-day course of 1,25(OH)_2_D_3_ does not lead to major changes in fexofenadine pharmacokinetics and in duodenal mRNA of OATP1A2, OATP2B1, P-gp, and MRP3.

Some limitations of this study have to be considered. First, we investigated healthy volunteers whose 1,25(OH)_2_D_3_ concentrations were at the lower range of normal, but who did not have manifest vitamin D deficiency. This may have prevented an effect of 1,25(OH)_2_D_3_ on folic acid pharmacokinetics becoming quantifiable. Second, a 10-day course of 1,25(OH)_2_D_3_ may have been too short to lead to substantial effects, although the induction was statistically significant in cell culture already after a 3-day treatment. Higher 1,25(OH)_2_D_3_ doses may have led to a visible effect on folic acid absorption and a statistically significant effect on fexofenadine pharmacokinetics. Last, since the variability in fexofenadine pharmacokinetics was higher than expected, a larger number of participants would have been necessary to unequivocally answer the question whether fexofenadine transport is significantly affected by 1,25(OH)_2_D_3_ treatment.

In summary, we show that a 10-day course of usual doses of 1,25(OH)_2_D_3_ does neither affect folic acid uptake nor fexofenadine pharmacokinetics in a significant manner. The results of this study underscore the importance of investigating whether in vitro findings can be confirmed in vivo.
